# Effects and mechanisms of APP and its cleavage product Aβ in the comorbidity of sarcopenia and Alzheimer’s disease

**DOI:** 10.3389/fnagi.2024.1482947

**Published:** 2024-11-25

**Authors:** Jiale Wu, Jun Tang, Di Huang, Yu Wang, Enyuan Zhou, Qin Ru, Guodong Xu, Lin Chen, Yuxiang Wu

**Affiliations:** Institute of Intelligent Sport and Proactive Health, Department of Health and Physical Education, Jianghan University, Wuhan, China

**Keywords:** sarcopenia, AD, myocerebral comorbidity, amyloid precursor protein, cleavage products, intervention

## Abstract

Sarcopenia and AD are both classic degenerative diseases, and there is growing epidemiological evidence of their comorbidity with aging; however, the mechanisms underlying the biology of their commonality have not yet been thoroughly investigated. APP is a membrane protein that is expressed in tissues and is expressed not only in the nervous system but also in the NMJ and muscle. Deposition of its proteolytic cleavage product, Aβ, has been described as a central component of AD pathogenesis. Recent studies have shown that excessive accumulation and aberrant expression of APP in muscle lead to pathological muscle lesions, but the pathogenic mechanism by which APP and its proteolytic cleavage products act in skeletal muscle is less well understood. By summarizing and analyzing the literature concerning the role, pathogenicity and pathological mechanisms of APP and its cleavage products in the nervous system and muscles, we aimed to explore the intrinsic pathological mechanisms of myocerebral comorbidities and to provide new perspectives and theoretical foundations for the prevention and treatment of AD and sarcopenia comorbidities.

## 1 Introduction

Sarcopenia is a syndrome characterized by a progressive and pervasive decline in skeletal muscle mass and muscle strength. In 2019, its definition was updated by the European Working Group on Sarcopenia in the Elderly in terms of application criteria and cutoff values (Van Ancum et al., 2020). The GLIS established the first global conceptual definition of sarcopenia in 2024. This definition encompasses three key components: muscle mass, muscle strength, and muscle-specific strength. These elements are considered essential for the diagnosis and understanding of sarcopenia, aiming to create a standardized framework for global research and clinical practice related to muscle health (Kirk et al., 2024). Sarcopenia reduces the quality of life of older people by leading to a variety of adverse outcomes, such as fractures, disability, frailty and increased risk of falls and death (Cruz-Jentoft and Sayer, 2019; Demurtas et al., 2020). The cost of medical care for older people with sarcopenia is more than five times the cost of health care for those without sarcopenia (Antunes et al., 2017), creating an enormous socioeconomic burden.

A growing body of epidemiological evidence suggests a potentially close relationship between sarcopenia and neurodegenerative diseases, such as AD (Sugimoto et al., 2016; Bozzi and Sciandra, 2020). The primary pathological causes of AD are amyloid plaques, resulting from the deposition of β-amyloid protein, and neurofibrillary tangles, which are linked to the abnormal hyperphosphorylation of tau protein. These two hallmark features contribute to the neurodegenerative processes that lead to cognitive decline and other symptoms of AD (Scheltens et al., 2021). Studies have shown that most people with sarcopenia also have AD, and most people with AD also experience muscle loss (Monteiro-Cardoso et al., 2015; Ogawa et al., 2018). At present, from the perspective of genetic factors, AD can be divided into sporadic AD and familial AD. Familial AD is a very rare autosomal dominant disorder with early onset caused by mutations in the amyloid precursor protein and presenilin genes. In contrast to familial forms, sporadic AD is very common, affecting more than 15 million people worldwide (Blennow et al., 2006). Existing reports have found more evidence of sarcopenia in sporadic AD (see Table 1), while there are few reports of familial AD and sarcopenia. The current literature suggests that sporadic AD accounts for the majority and may be more susceptible to sarcopenia due to complex interactions between aging and environmental factors (Marras and Goldman, 2011). Therefore, this article focuses on the relationship between sporadic AD and sarcopenia comorbidity.

**Table 1:** Supplementary evidence that AD is associated with sarcopenia.

	Cohort study methods	Study population	Sample size	Evaluation index	Conclusion	Year	PMID
1	Cross-sectional study	65–89 years old	205	AD: MMSE: Score ≥ 21 Sarcopenia: Low HGS: < 6 kg for men; < 8 kg for women Slow gait speed: < 0.8 m/s Low muscle mass: < 7.0 kg/m^2^ for men; < 5.7 kg/m^2^ for women	Appetite could be a modifiable risk factor for sarcopenia in patients with MCI and early-stage AD.	2018	30619874
2	Cross-sectional study	≥75 years old	285	AD: MMSE: early AD: score ≥ 24; mild AD: score: 21–23; moderate AD: score ≤ 20 Sarcopenia: Low HGS: < 26 kg for men; < 18 kg for women Low gait speed: ≤ 0.8 m/s Low muscle mass: SMI: 7.0 kg/m^2^ for men; 5.7 kg/m^2^ for women	Although muscle functions and physical performance decrease with aging, these functions further decrease in the early stage of AD. Subjects with AD, even the early stages of AD, showed a high prevalence of sarcopenia.	2018	30210435
3	Cross-sectional study	≥50 years old	496	AD: MMSE: Score < 24 Sarcopenia: Gait speed test: ≤ 0.8 m/s Low SMI: ASM SMI Slow gait speed Low HGS: < 30 kg for men; < 20 kg for women	Significant longitudinal associations were observed between sarcopenia, mild cognitive impairment and cognitive function among older Mexican adults.	2021	34535964
4	Cross-sectional study	The average participant was 80.9 years old	1,175	AD: MMSE Sarcopenia: SMI Low muscle mass: < 8.87 for men; < 6.42 for women Low HGS: <30 kg for men < 20 kg for women	Poor muscle function, but not reduced lean muscle mass, drives the association of sarcopenia with late-life cognitive impairment.	2021	33954985
5	Cross-sectional study	65–89 years old	497 MCI 684 AD patients	AD: ADAS-cog: Score ≤ 70 Sarcopenia: Low HGS: < 18 kg for women; < 28 kg for men Low SMI: < 5.7 kg/m^2^ for women; < 7.0 kg/m^2^ for men Low physical performance: TUG ≥20 s	The specific association of sarcopenia and its components with cognitive domains may provide the key to elucidating the muscle–brain interactions in AD.	2022	35441196
6	Cross-sectional study	≥60 years old	23,694	AD: MMSE: Score > 13 Sarcopenia: Low HGS: < 27 kg for men; < 16 kg for women Slow sit-to-stand test: > 15 s	The results suggest that sarcopenia is the major predictor of screen-detected mild cognitive impairment in older adults, not overweight or obesity.	2022	36330135
7	Cross-sectional study	≥65 years old	130	AD: Diagnostic and Statistical Manual of Mental Disorders Sarcopenia: Low SMI: ≤ 8.87 kg/m^2^ for man; ≤ 6.42 kg/m^2^ for woman Low HGS: ≤ 30 kg for men ≤ 20 kg for women	Sarcopenia has a high prevalence in dementia.	2022	36114480
8	Cross-sectional study	≥70 years old	45	AD: MMSE CDR Mild: CDR 1; Moderate: CDR 2; Severe: CDR 3 Sarcopenia: DEXA: > 28% for men; > 39% for women ASMI: < 7.2 for men < 5.7 for women SO: Residual values: < -2.3 for men; < -3.4 for women	Women with AD had higher body fat and lower muscle mass than men. SO occurs in older women with AD. Men with higher body fat showed better cognitive performance, independent of age and education.	2022	35719255
9	Cross-sectional study	≥65 years old	201	AD: SMMT and SMMT-E: Normal cognitive function: Score ≥ 24 points, Mild dementia: Score: 18–24, Severe dementia: Score ≤ 18 Sarcopenia: Low HGS < 27 kg for men < 16 kg for women Low SMI: < 37% for men < 27.6% for women 4 m walking speed test: ≤ 0.8 m/s	The rate of sarcopenia was significantly higher in older individuals with cognitive than those with normal cognitive functions.	2022	35569922
10	Cross-sectional study	≥65 years old	56	AD: MMSE Sarcopenia: Frailty: FRAIL ≥1 CFS > 3 Low HGS: < 16 kg for men; < 27 kg for women Low physical performance: TUG: ≤ 0.8 m/s 5 × SST: > 15 s	We found that falls were not influenced by AD stage. Both physical and cumulative frailty were strongly associated with falls in patients with mild-to-moderate AD.	2023	36647232

SMI, Skeletal muscle index; TUG, Timed up and go; HGS, Hand grip strength; ADAS-cog, AD Assessment scale-cognitive subscale; ASM, Appendicular skeletal muscle mass; ASMI, Appendicular skeletal muscle mass index; MMSE, Mini-Mental State Examination; FRAIL, Fatigue, resistance, ambulation, illness and loss of weight; CFS, Clinical frailty scale; 5 × SST, Five-times sit-to-stand test; SMM, Skeletal muscle mass; SO, Sarcopenic obesity; CDR, Clinical dementia rating; DEXA, Dual-emission X-ray absorptiometry; SMMT, Standardized mini-mental test; SMMT-E, Standardized mini-mental test for the untrained.

The nerve center can regulate the function of muscle contraction and stretching. On the other hand, the retrograde signal generated from the muscle can also affect the function of neurons (Kennouche et al., 2022), indicating that there is a close relationship between the nervous system and the muscles; however, the common pathological mechanism between these two systems has not been extensively studied. The cascade effects caused by APP and its proteolytic cleavage products, which are present in the muscle and nerve centers as well as at the NMJ, are breakthrough points for the exploration of the pathological mechanism of muscle–brain comorbidity (Nogalska et al., 2010; Hu et al., 2018; Wu et al., 2023).

Although tau protein is present in both the brain and muscle, this review focused on the effects of Aβ (amyloid-beta). First, the accumulation of Aβ plaques is considered a more central pathological hallmark of AD. Second, research on Aβ began earlier, and its significance in AD and other neurodegenerative diseases has been widely validated. In comparison, while tau protein has also attracted attention for its role in disease, its involvement in muscle–brain comorbidity mechanisms has not been studied as extensively as Aβ is. Finally, tau pathology is characterized primarily by abnormal phosphorylation, leading to the loss of microtubule stability in neurons, which results in neuronal dysfunction and cell death. This mechanism is more directly linked to neurodegenerative processes, whereas its specific effects on muscle cells require further evidence. Notably, tau pathology is another important issue to be closely followed in our future research.

Therefore, in this review, we describe the current status of knowledge about sarcopenia and AD comorbidity, the physiological and pathological functions of APP cleavage products, and the potential role and mechanism of cleavage products in the development of sarcopenia and AD. We also summarize the existing intervention methods based on splicing product clearance, providing new ideas and a theoretical basis for the clinical prevention and treatment of sarcopenia and AD comorbidity in the future.

## 2 Comorbidity status of sarcopenia and AD

The word “sarcopenia” originates from the Greek language to describe the reduction and deprivation of the physical body and has evolved to describe the age-related decline in muscle composition and function (Rosenberg, 1997). Sarcopenia is a progressive and systemic skeletal muscle disease. It is a syndrome characterized by a loss of skeletal muscle mass and strength (Cruz-Jentoft et al., 2019). The estimated prevalence of sarcopenia worldwide ranges from 10 to 16% (Beaudart et al., 2019). According to the EWGSOP, the prevalence ranges from 8 to 36% in those under 60 years of age and from 10 to 27% in those over 60 years of age (Petermann-Rocha et al., 2022). A study of 6,172 participants aged 60–94 years revealed that the prevalence of possible sarcopenia, sarcopenia and severe sarcopenia in China were 38.5, 18.6 and 8.0%, respectively, and that Chinese rural elderly individuals were more likely to have sarcopenia than urban elderly individuals were (Wu et al., 2021). Clinically, sarcopenia is characterized by reduced skeletal muscle mass and muscle function, such as reduced functional motor units (mainly type II muscle fibers), altered levels of anabolic hormones, and decreased protein synthesis (Argilés et al., 2015). Grip strength (using a Jamar dynamometer) and the chair standing test (the number of times the patient can stand up and sit in a chair in a 30-s interval) are commonly used for initial diagnosis in clinical practice and research (Van Ancum et al., 2020). The diagnostic criteria are as follows: grip strength in men <27 kg, grip strength in women <16 kg (Dodds et al., 2014), and able to stand five or more times in 15 s (Cesari et al., 2009). To objectively evaluate low muscle mass and quantity in patients for further diagnosis of sarcopenia, the EWGSOP recommends the use of DXA, BIA, CT, or MRI, of which CT and MRI are considered the gold standards for noninvasive assessment of muscle quantity/quality (Rossi et al., 2014; Beaudart et al., 2016; Buckinx et al., 2018).

Patients with sarcopenia often suffer from falls, fractures, weakness and even death due to loss of muscle strength, which greatly affects the normal life of elderly individuals (Cruz-Jentoft and Sayer, 2019). Many factors affect sarcopenia, such as aging, lack of physical activity, insufficient nutritional intake, and the influence of other diseases, among which lack of physical activity is considered to be the main risk factor. Studies have shown that for every additional hour of sedentary time in elderly individuals, the risk of sarcopenia increases by 33% (Gianoudis et al., 2015). However, the intrinsic cause of sarcopenia is the disruption of the balance between muscle protein synthesis and degradation. Many factors regulate muscle protein synthesis and degradation, including the inflammatory response, oxidative stress, and hormonal regulation. For example, inflammatory factors (TNF-*α*, IL-6, and IL-1) promote the infiltration of inflammatory cells by activating the NF-κB pathway, causing muscle damage (Zhang et al., 2022). Oxidative stress attenuates muscle protein anabolism and enhances catabolism (Koopman and van Loon, 2009), and elevated levels of chronic low-grade inflammation induced by oxidative stress are detrimental to skeletal muscle (Howard et al., 2007). Myostatin inhibits the Akt/mTOR axis, increases FOXO activity, and induces the expression of MURF-1 and MAFbx to promote muscle protein degradation (Wiedmer et al., 2021).

The treatments for sarcopenia can be divided into drug treatment and nondrug treatment. Drug therapy includes growth hormone, myostatin inhibitors, and troponin activators. However, the efficacy is not ideal (Cho et al., 2022). Nondrug treatment is based mainly on diet and exercise interventions. Studies have shown that different forms of exercise play positive roles in muscle mass, strength and physical performance, among which resistance exercise has been used as the main treatment for sarcopenia (Hurst et al., 2022; Sayer and Cruz-Jentoft, 2022).

AD, the prevalent cause of dementia, is a process of cognitive decline associated with age and specific neuropathology (Soria Lopez et al., 2019). With the serious aging of the world’s population, the incidence of AD is increasing (Weller and Budson, 2018). There are currently 44 million people in the world who suffer from dementia. By 2050, there will be 152 million AD patients worldwide and 28 million AD patients in China. In addition, the mortality rate of AD patients is high, and AD is officially ranked as the sixth cause of death in the United States (Alzheimer’s Disease Facts and Figures 2024). The average survival time of AD patients in China is 5.9 years. Clinically, AD patients may experience emotional changes such as anxiety and irritability. In the late stage of the disease, patients may experience severe memory loss, loss of speech function and motor function decline (Zis and Strydom, 2018). Patients with AD often experience memory loss, cognitive impairment and loss of ability to perform ADLs. Depressive symptoms have been reported in 20–30% of patients with early AD (Kalia, 2003).

The main pathological features of AD are the accumulation of extracellular Aβ, which results in the formation of senile plaques, and the abnormal phosphorylation of intracellular tau protein, which leads to neurofibrillary tangles (Rostagno, 2022). PET and CSF protein analysis were used to detect the contents of the Aβ and tau proteins in the cerebrospinal fluid of patients to determine whether they are at risk of AD (Mantzavinos and Alexiou, 2017; Scheltens et al., 2021). At present, the diagnostic criteria for AD have been constantly updated on the basis of biomarker contributions to improve AD diagnostic accuracy and pharmacological intervention. Patients diagnosed with pathological evidence of Aβ or Tau are considered to be at risk for AD (Jack et al., 2011).

In summary, amyloid plaques, neurofibrillary tangles, synaptic dysfunction leading to neurotransmitter imbalance, neuroinflammation, gut microbial destruction, oxidative stress and impaired autophagy all contribute to the pathogenesis of AD (Khan et al., 2020). For example, amyloid β plaque formation is due to abnormal cleavage of APP caused by mutations in the APP, PS1, PS2, and APOE4 genes, leading to the accumulation of Aβ42 (O'Brien and Wong, 2011). Excessive activation of CDK5 leads to the hyperphosphorylation of the tau protein, which reduces its affinity for microtubules, resulting in the formation of neurofibrillary tangles and loss of the ability to maintain the structure of nerve cells (Crews and Masliah, 2010). The neurotransmitter 5-hydroxytryptamine regulates cortical plasticity and memory, and this dysfunction leads to memory loss (Vakalopoulos, 2017). Proinflammatory cytokines in the brain cause damage to dendritic spines and impede the clearance of Aβ (Calsolaro and Edison, 2016). Distruption of the gut microbiota leads to increased secretion of cytotoxic bile acids, which cross the BBB and are deposited in the brain, leading to the generation of ROS and inflammation, as well as nerve cell degeneration (MahmoudianDehkordi et al., 2019); altered intestinal microflora activity and increased nitrate intake can lead to overproduction of NO, which can cause axonal degeneration, neuroinflammation and neurodegenerative disease (Luca et al., 2019). Oxidative stress promotes the excessive production of ROS, leading to increased cell permeability and calcium influx, which promote ROS accumulation. Moreover, Aβ plaques are more difficult to remove after oxidation (Luca et al., 2019). Oxidative stress, neurofibrillary tangles and presenilin-1 can lead to autophagy dysfunction, which accelerates the AD process (Funderburk et al., 2010). A variety of drugs have been developed for the treatment of AD patients, but their effects are not satisfactory and are accompanied by severe side effects. Interestingly, some studies have shown that physical exercise has good preventive and therapeutic effects on AD (De la Rosa et al., 2020), which will be described in the second half of the article.

Sarcopenia is closely related to AD (see Table 1). Many studies have reported that sarcopenia is associated with Alzheimer’s dementia, mild cognitive impairment, and cognitive decline. On the one hand, patients with AD often experience symptoms of muscle loss (Bramato et al., 2022). Defects in skeletal muscle may be an early sign of AD. Johnson et al. (2006) studied whether weight loss occurred before the diagnosis of AD and reported that, compared with healthy subjects, AD patients lost weight at twice the rate of normal subjects in the year before diagnosis, suggesting that the loss of skeletal muscle may be an early symptom of AD. A study of muscle function at different stages of AD revealed that although muscle strength decreased in early AD patients, there was no loss of muscle mass. Patients with moderate-stage AD have a decrease in muscle mass and strength, and patients with late-stage AD have a slow gait speed. This study also revealed that the prevalence of sarcopenia is still high even in the early stage of the disease (Ogawa et al., 2018; Waite et al., 2021). Other studies have shown that the risk of sarcopenia in AD patients is greater than that in patients without cognitive impairment, and the symptoms of sarcopenia in patients with advanced AD are more severe than those in patients with sarcopenia but without cognitive deficits (Soto et al., 2012). In addition, AD pathology and cerebral amyloid angiopathy are associated with more rapid motor decline, increasing the risk for sarcopenia (Buchman et al., 2020).

However, patients with sarcopenia often present with cognitive deficits. Sarcopenia is associated with a greater probability of AD than individuals without sarcopenia, and reduced muscle function is associated with cognitive impairment later in life (Beeri et al., 2021). In a study of healthy older adults in more than 900 communities followed for 3.6 years, proportional hazards models adjusted for age, sex, and education revealed that lower muscle strength was associated with a greater risk for AD (Boyle et al., 2009). In another study of grip strength in patients with AD, the risk of AD increased by 1.5% for each pound decrease in grip strength (Buchman et al., 2007). These findings suggest that decreased muscle strength may contribute to an increased risk of AD. In addition, increasing evidence suggests that myokines can directly affect brain function (Scisciola et al., 2021). For example, FNDC5 can improve synaptic plasticity and memory deficits in AD (Lourenco et al., 2019). Healthy muscle status is beneficial to cognitive function in AD patients. An experiment in AD mice revealed that the cognitive deficits of AD mice were positively correlated with increased expression of myostatin. The inhibition of myostatin expression in AD mice increased muscle mass and strength and improved cognitive ability (Lin et al., 2019).

An increasing life expectancy and a growing elderly population are likely to lead to an accompanying increase in the prevalence of sarcopenia and AD in the coming decades (Dao et al., 2020; Fan et al., 2022). However, the mechanisms of sarcopenia and cognitive impairment are complex. Both AD and sarcopenia share similar environmental risk factors and follow similar pathological processes, such as aging, chronic inflammation, oxidative stress, and mitochondrial dysfunction (Migliavacca et al., 2019). Therefore, it is particularly important to explore the molecular mechanisms underlying the comorbidity of sarcopenia and AD and to explore intervention methods.

The accumulation of APP and its proteolytic cleavage product Aβ *in vivo* and *in vitro* is toxic to muscle, the central nervous system and other cells (Tsoy et al., 2024). Elderly plaques formed by Aβ accumulation in the brain have been proven to be one of the initial causes of AD, and the skeletal muscle of patients with sarcopenia also seems to produce a variety of misfolded proteins. Among these proteins, the abnormal expression of APP and the aggregation of its proteolytic cleavage product may play key roles, resulting in a series of physiological and biochemical reactions, eventually leading to the occurrence of sarcopenia (Jimenez-Gutierrez et al., 2022). Previous studies have confirmed that high skeletal muscle mass is inversely associated with the risk of positive Aβ expression (Kang et al., 2022). In the following, after a brief introduction to APP and its proteolytic cleavage product, we describe the effects and mechanisms of APP and its proteolytic cleavage product Aβ in sarcopenia and AD.

## 3 The origin and route of Aβ

### 3.1 The structure of APP

**Figure 1:**
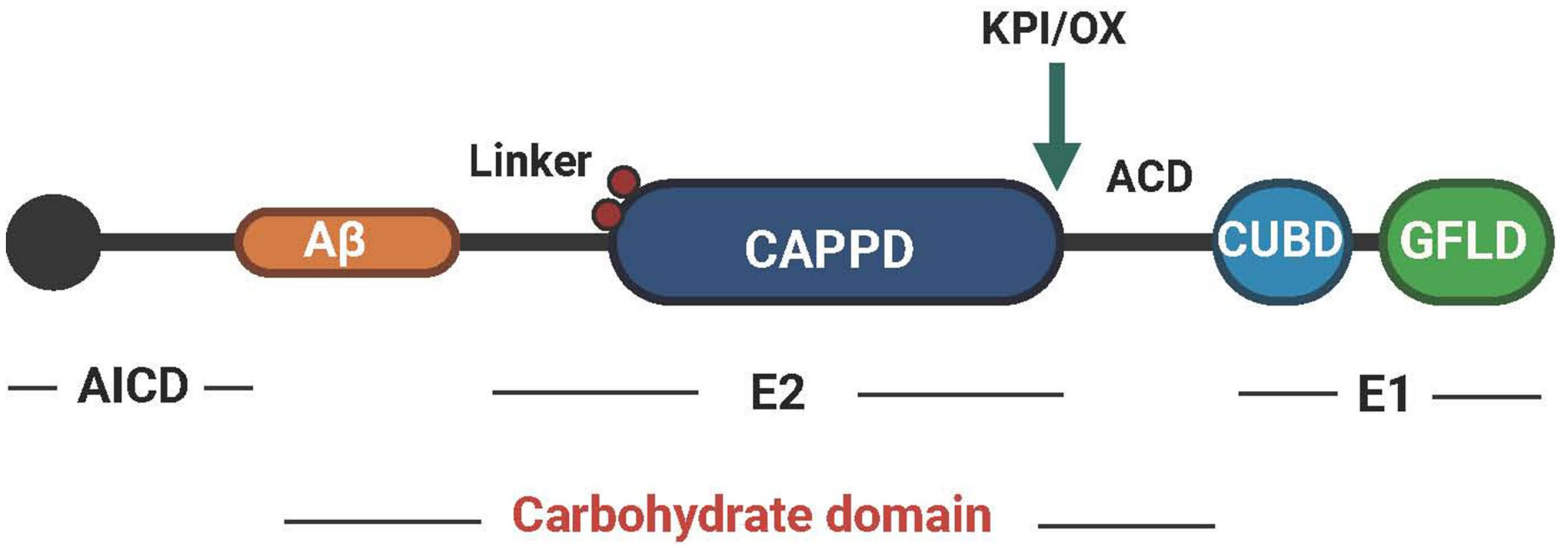
The structural pattern diagram of APP. APP can be divided into three regions, AICD, E2 and E1, and each region performs different functions. Created with BioRender.com.

APP is a type I transmembrane protein encoded by a gene located on chromosome 21 (Wang et al., 2017). Its molecular structure has a large extracellular glycosylation N-terminal domain and a short cytoplasmic C-terminal domain. The structure of human APP695 is shown in Figure 1. The cysteine-rich globular domain (E1 region) consists of an N-terminal GFLD and a CUBD. This region can promote synapse growth and activate MAPK. It also provides a binding site for APP dimerization (Baumkötter et al., 2012). The E1 region is connected to the carbohydrate domain by an ACD. The carbohydrate domain can be divided into an E2 domain, also called CAPPD, and a connecting domain (also called the juxtamembrane domain), which contains two glycosylation sites in the extracellular domain (red spheres). The E2 domain plays a role in promoting synaptic growth and cell adhesion (Wilkins and Swerdlow, 2017). The carbohydrate domain is followed by the transmembrane domain and the AICD. The AICD domain can be posttranslationally modified and plays a critical role in APP trafficking, endocytosis and intracellular signaling pathways through its YENPTY motif (Müller et al., 2017). The KPI (present in APP770 and APP751) domain is above the insertion site with the Ox-2 domain (present in APP770) (Reinhard et al., 2005).

### 3.2 Hydrolysis of APP

**Figure 2:**
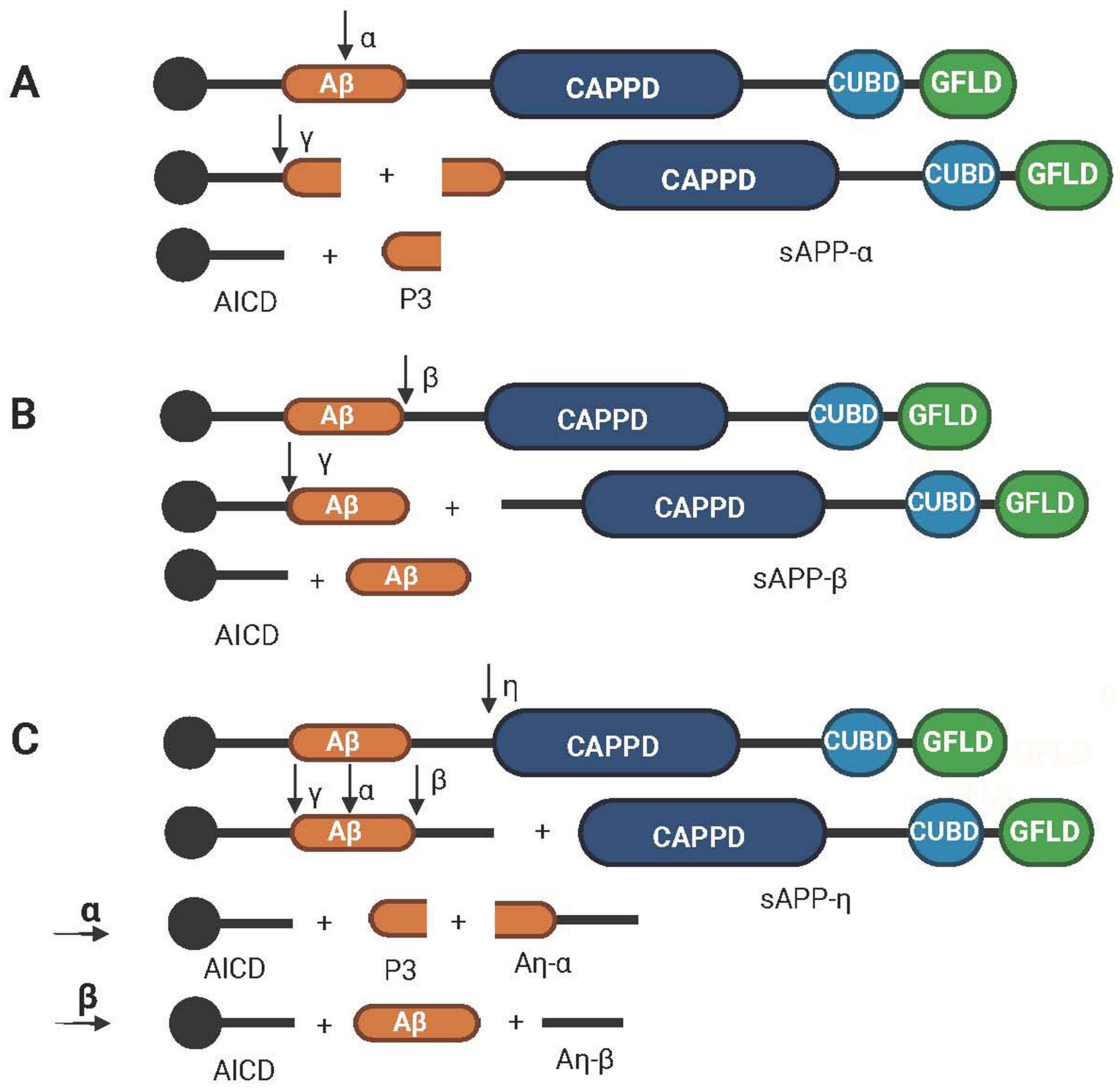
Schematic diagram of the hydrolysis path of APP. (A) The nonamyloid protein pathway, which is cleaved sequentially by *α* secretase and γ secretase to obtain AICD, P3 and sAPPα. (B) The amyloid protein pathway, which is cleaved sequentially by β-secretase and γ-secretase to obtain AICD, Aβ and sAPPβ. (C) The third pathway of APP hydrolysis, which involves cleavage by *η*, α, β and γ secretases in turn to obtain AICD, P3, Aβ, Aη-α, Aη-β and sAPP-η. Created with BioRender.com.

APP hydrolysis can be divided into three pathways. The first two hydrolysis pathways are distinguished by the types of cleavage enzymes and different cleavage sites. Compared with the first two pathways, the third pathway involves different cleavage enzymes, frequencies and sites as shown in Figure 2.

The first pathway is called the nonamyloid pathway, and its cleaving enzyme is alpha-secretase, which divides APP into an *α*-App fragment and a membrane-linked CTF. The former has neuroprotective functions (Chasseigneaux and Allinquant, 2012). The CTFs are then cleaved via *γ*-secretase to produce the P3 peptide and the AICD (Dunot et al., 2023). Meanwhile, the P3 peptide is released into the extracellular space, and AICD is retained in the cytoplasm (Kalia, 2003) as shown in Figure 2A. The second pathway is called the amyloid formation pathway, in which APP is divided into β-APP (N-terminal fragment) and a C-terminal fragment by β-secretase and then undergoes cleavage by γ-secretase to produce Aβ and AICD as shown in Figure 2B. During cleavage, γ-secretase can produce APP to amyloid peptides of different chain lengths, including Aβ37, 38, 39, 40, 42, and 43 (Bibl et al., 2006; Bibl et al., 2007). Aβ40 and Aβ42 are the two main sources of Aβ in the brain. Although soluble Aβ40 is much more abundant than soluble Aβ42 is, it tends to aggregate because of the hydrophobic nature of the two terminal residues of Aβ42 (Guo et al., 2020). Aβ42, the main material of plaques produced by amyloid deposition and one of the main pathological features of AD, is neurotoxic (Oakley et al., 2006). Aβ usually exists as a monomer of <10 kDa as well as an oligomer of >100 kDa (Tolar et al., 2024). oAβ is more toxic than Aβ is, and oligomers of Aβ42 have been shown to inhibit neuronal viability by up to 10-fold compared with monomeric Aβ (Huang and Liu, 2020). In addition, Aβ38 levels, Aβ42 levels, and the Aβ42/Aβ38 ratio in CSF have been shown to be the best indicators for distinguishing AD from DLB (Mulugeta et al., 2011). The third path is different from the above two paths; the APP is first divided into CTF*η* and η-APP fragments by the η secretase. After that, it passes through the first two paths again and is cleaved continuously by α, β, and γ secretases. The final fragment will form one more amyloid protein, Aη-α or Aη-β, than the fragments obtained via the first two paths (Hefter et al., 2020) as shown in Figure 2C.

### 3.3 The clearance of Aβ

The level of Aβ depends on whether the production, degradation and clearance of Aβ are balanced. Aβ in ISF can be cleared directly from the brain parenchyma, and the Aβ in ISF can be transported to the blood for degradation across the BBB or by vascular wall cells (Zhao et al., 2018). It can also be cleared through the circulation and lymphatic system as the ISF flows into the CSF (Tarasoff-Conway et al., 2015). The main mechanism includes the clearance effect mediated by receptors (LRP1 and LDLR) on cells (nerve and glial cells) in the brain parenchyma, and vascular epithelial cells are degraded from perivascular uptake or proteolysis promoted by endopeptidases (NEP and IDE) (Liu et al., 2013). In addition, after lipidation of the apoproteins, the lapidated APOE binds to Aβ through cell surface receptors and plays a role in clearance (Lanfranco et al., 2020). However, APOE also binds to astrocyte receptors and forms a competitive relationship with Aβ, which reduces the binding of Aβ to astrocyte receptors and thus inhibits the clearance of Aβ (Verghese et al., 2013). It has been suggested that Aβ binding to HPSGs on nerve cells also hinders Aβ hydrolysis and promotes Aβ aggregation and oligomerization (Liu et al., 2016).

## 4 Effects of Aβ in the brain

### 4.1 Effects of Aβ on neurons

Aβ can affect cell homeostasis by regulating MERCS, destroying the mitochondrial structure, causing defects in mitophagy, and promoting neuronal death (Leal et al., 2020). High levels of Aβ bind to mitochondrial outer membrane protein (VDAC1) and terminate mitochondrial protein trafficking, thereby preventing cytochrome c from binding to cytochrome oxidase and causing toxicity (Devi et al., 2006; John and Reddy, 2021). Abnormal accumulation of Aβ inhibits autophagy and mitophagy in the hippocampus of APP mice, thereby hindering the clearance of fragmented mitochondria and promoting neuronal death (Manczak et al., 2018). In addition, the intracellular accumulation of Aβ42 disrupts Akt signaling, possibly by disrupting the interaction between Akt and its activator PDK-1 (Querfurth et al., 2022). Downregulation of Akt increases GSK-3β activity and promotes neuronal cell death (Lai et al., 2023). Phosphorylation of GSK-3β can also inhibit the activity of HSF-1 to produce a stress response in nerve cells (Eldar-Finkelman and Martinez, 2011). In addition, downregulation of PI3K-Akt increased the production and decreased the clearance rate of Aβ (Dong et al., 2012).

Excessive accumulation of Aβ can lead to an inflammatory response. Aβ induces neuroinflammatory responses by disrupting mitochondrial dynamics. The interaction between mitochondrial fission proteins such as Drp1 and Aβ results in increased activity of Drp1, which leads to excessive fragmentation of mitochondria and aggravates mitochondrial dysfunction (Reddy et al., 2018). This induces the activation of the NLRP3 inflammasome and leads to the release of the inflammatory factor IL-1β (Sbai et al., 2022). In addition, Aβ can induce pyroptosis in nerve cells at the cellular level. Aβ induces pyroptosis through the GSDMD protein, which is cleaved to form the P30 terminus by caspase-1 (NLRP3-caspase-1 signaling is an important signal that mediates GSDMD cleavage). GADMD located on the cell membrane opens membrane channels and releases inflammatory cytokines. One study also revealed that the inhibition of pyroptosis could alleviate nerve damage and play a role in alleviating cognitive ability in AD (Han et al., 2020). Aβ can also induce MC65 cells to produce a strong proinflammatory response and release inflammatory factors by activating RAGE, which further aggravates the aggregation and toxicity of Aβ (Currais et al., 2016).

Excessive accumulation of Aβ can lead to an oxidative stress response. Aβ can inactivate ABAD by preventing NAD^+^ from binding to ABAD, which leads to damage to mitochondrial membrane permeability and respiratory enzymes and eventually leads to mitochondrial failure (Morsy and Trippier, 2019). Mitochondrial dysfunction reduces the production of the metabolite acetyl-CoA, leading to oxidative stress/ferroptosis. This finding was supported by previous studies showing that the level of acetyl-CoA was decreased in HT22 neurons under oxidative stress/ferroptosis (Currais et al., 2019). In addition, excessive Aβ increases ROS production by inhibiting *α*-ketoglutarate dehydrogenase activity and by disrupting the electron transport chain. An abnormal increase in ROS leads to excessive lipid oxidation and DNA damage to mitochondria (Tönnies and Trushina, 2017). An increase in ROS also leads to a decrease in the calcium uptake capacity of mitochondria, which leads to synaptic damage (Lee S. H. et al., 2012). As ROS reduce the capacity of mitochondria to take up Ca^2+^ autonomously, they compensate for the Ca^2+^ released from the ER of cortical neurons to the cytoplasm as a result of Aβ stimulation. Moreover, these calcium ions can also cause direct damage to mitochondria, which again leads to the aggravation of ROS production, the transmission of apoptotic signals, and the induction of nerve cell death (Calvo-Rodriguez et al., 2020).

In addition to mitochondria, low-molecular-weight Aβ peptides have been suggested to interrupt mitochondrial–ER anchoring, thereby causing ER collapse (Lai et al., 2009). Reduced Aβ is associated with reduced ER stress. Aging PS2 mutant mice exhibited suppressed BACE1 activity and C99 content in brain tissue during treadmill exercise, along with downregulation of GRP78 and PDI (GRP78 is a marker of the ER stress response) (Endres and Reinhardt, 2013). In addition, studies have shown that the accumulation of oAβ can also induce oxidative stress in the ER (Endres and Reinhardt, 2013; Kondo et al., 2013). For example, GRP78 and XBP-1 protein levels are increased in Aβ-treated neurons (Costa et al., 2013), and ER stress can lead to an imbalance in the concentration of calcium ions stored in the ER, leading to nerve cell death (Endres and Reinhardt, 2013).

The abnormal expression of Aβ can also affect synaptic plasticity. Aβ can interact with a variety of proteins involved in synapse formation. The sites of action of Aβ are evenly distributed across the presynaptic and postsynaptic membranes (Wang et al., 2009). Aβ binds to a variety of receptors on the postsynaptic membrane, such as NMDAR, EPHB2, PrPc, EPHA4, and LiLRB2. The binding of these receptors to Aβ results in synaptic toxicity. Aβ can bind to NMDA receptors, resulting in abnormal calcium metabolism, causing oxidative stress, free radical production, and neuronal loss. Fyn is an important regulator of NMDAR-mediated oAβ neurotoxicity (Guo et al., 2020). Studies have shown that Aβ accumulative AD mice exhibit impaired synaptic transmission and defective LTP even before Aβ accumulation forms plaques (Oddo et al., 2003), mainly because Aβ affects calcium channel activity and glutamate receptor-dependent signaling pathways (Rajmohan and Reddy, 2017). The mechanism involves Aβ binding to the 7*α*-nicotinic acetylcholine receptor, which reduces calcium influx, leading to the internalization of NMDA receptors and the enhancement of LTD (Koffie et al., 2011). At the same time, Aβ activates mGLuRs, calcineurin, the Caspase-3 pathway and AMPAR and causes AMPAR internalization, leading to synaptic damage. Among them, calcineurin can also activate the NFAT pathway and the STEP pathway, causing synaptic spine loss and leading to LTD enhancement through dephosphorylation of the NR2B subunit of the NMDA receptor (Koffie et al., 2011). In addition, disruption of the PI3K–Akt signaling pathway caused by excessive Aβ affects synaptic plasticity and LTP expression (Long et al., 2021). Finally, in the early stage of AD, Aβ42 upregulates the expression of the VGLUT1 protein, which is responsible for transporting Glu to synaptic vesicles, increasing the storage and release of Glu from vesicles, leading to transient hyperexcitability of neurons and causing excitatory damage to neurons. During disease development, Aβ42 induces the overexpression of the spontaneous release-related protein Vti1a, which leads to high-frequency spontaneous vesicle exocytosis of Glu. Along with the downregulation of VGLUT1 protein expression, the content and release efficiency of Glu in vesicles are significantly reduced, which leads to the desensitization of NMDA receptors and eventually leads to neuronal degeneration (Yang et al., 2023).

### 4.2 The effect of Aβ on microglia

Microglia, a type of glial cell, are mononuclear phagocytes that play important roles in the maintenance of the central nervous system. Microglia can secrete glial and neurogenic neurotrophic factors that help damaged neurons recover and can also help the brain remove unwanted metabolites (Nayak et al., 2014). However, microglia are often activated in the early stage of AD and lose their recovery ability, mainly because of the accumulation of oAβ, the decomposition product of APP, and the long-term damage caused by inflammatory factors, which may also be induced by oAβ. Continuous stimulation of microglia leads to nerve degeneration (Fan et al., 2017). Microglial activation can be divided into M1- and M2-type microglia, and M2-phenotype microglia are widely considered to have neuroprotective effects. Specifically, Aβ binding to TREM2 activates the SYK pathway through DAP12, an adaptor protein of TREM2, leading to Aβ degradation. In contrast, the activation of microglia induced by excess Aβ belongs to the M1 type (proinflammatory phenotype), which induces neuronal damage (Nakagawa and Chiba, 2015). The binding of Aβ to different microglia receptors, including TREM2, TLR4, CD36, LRP1, and RAGE, simultaneously activates a number of inflammatory pathways, including the NF-kB, JAK–STAT, and NLRP3 inflammasome pathways. The expression of proinflammatory mediators, such as TNF-α and the interleukins IL-1, IL-6, IL-12, IL-17, and IL-18, the IL-23 cytokines CCL12 and CXCL10, and other proinflammatory mediators, as well as ROS, RNS, iNOS and COX-2 (Li et al., 2004; Chauhan et al., 2021), is also increased, leading to neuroinflammation and neurotoxicity. By interfering with the phagocytosis and clearance of Aβ, the accumulation of Aβ damages the brain, forming a vicious cycle.

Chemokines guide microglia and astrocytes to the site of neuroinflammation and aggravate local inflammation (Domingues et al., 2017). In addition, the accumulation of Aβ causes damage to the mitochondria of microglia and further activates the release of inflammatory cytokines (Xiao et al., 2022). Other studies have shown that activated microglia can also affect the normal physiological function of astrocytes. For example, activated microglia can transform normal astrocytes into A1-type toxic astrocytes through the production of proinflammatory factors (Liddelow et al., 2017), thus accelerating the release of inflammatory factors and worsening the disease.

### 4.3 The effect of Aβ on astrocytes

Astrocytes play an important role in the brain. Astrocytes can provide nutrients for neurons to maintain normal life and play a role in the repair of brain damage and the maintenance of the extracellular ion balance in a calcium-dependent manner (Sofroniew and Vinters, 2010). The effects of Aβ on astrocytes are reciprocal. Excessive Aβ production can change the structure and function of astrocytes and damage their protective effect on nerve cells (Shrivastava et al., 2013). The detrimental effects of astrocytes in AD can also exacerbate the accumulation of Aβ, leading to the rapid formation of amyloid plaques (Cai et al., 2017). Astrocytes increase Aβ levels mainly by promoting Aβ production and inhibiting Aβ clearance. On the one hand, Aβ causes astrocyte dysfunction, which leads to a decrease in Aβ clearance efficiency (Kuruppu et al., 2017). On the other hand, Aβ induces the activation of astrocytes, also known as reactive astrocytes, which can produce oxidative stress, inflammatory factors, chemokines and cytotoxicity; promote the production and accumulation of Aβ; and cause damage to neurons (Choi et al., 2016). Specifically, oAβ can activate astrocytes directly through α7-nAchRs, CaSR, CD36, CD47 and AQP4. Reactive astrocytes interfere with synapse development and axon growth through proinflammatory cytokines, reactive oxygen species and reactive nitrogen species, thereby increasing inflammation and oxidative stress. At this time, there is also an effect on the drainage of interstitial fluid and Aβ phagocytosis by microglia (Lian et al., 2016; Ahmed et al., 2017).

**Figure 3:**
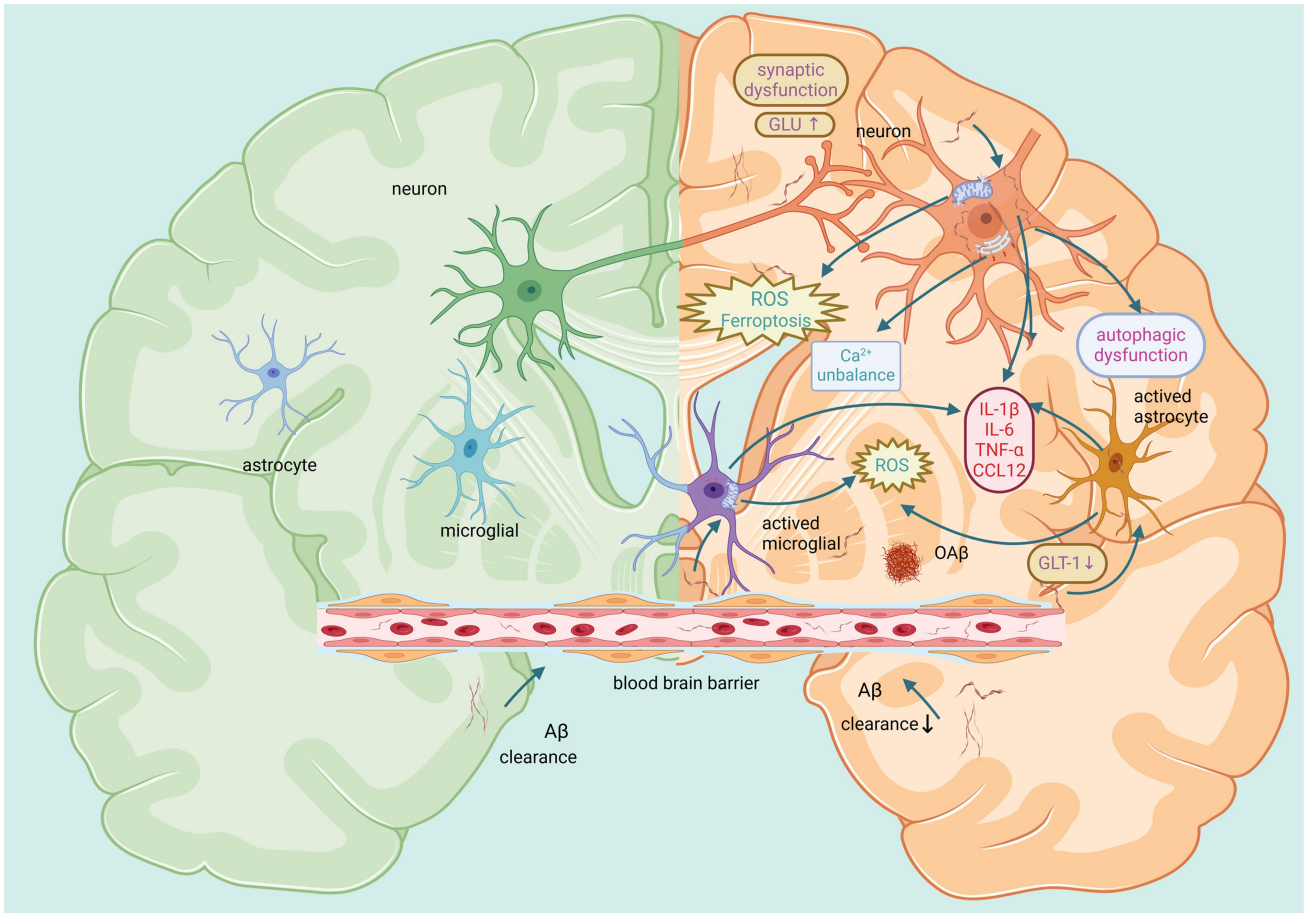
Main effects of Aβ on neurons, microglia, and astrocytes in the brain. The left panel represents a healthy brain with small amounts of Aβ. The right panel shows that large amounts of Aβ induce adverse reactions such as autophagic dysfunction, inflammation, oxidative stress, calcium imbalance, and impaired synaptic function. This affects the function of neurons and glial cells, ultimately causing harm to the brain. Created with BioRender.com.

Aβ also reduces the distribution of GLT-1 on the surface of astrocytes. It leads to dysregulation of extracellular glutamate homeostasis and indirectly induces glutamatergic toxicity (Scimemi et al., 2013). Notably, the BBB is dynamically maintained by astrocytes, pericytes and BECs. Therefore, astrocyte dysfunction prevents the transport and clearance of Aβ through the BBB and continues to cause damage to the reticular structure of the BBB (Thomas et al., 2017). Moreover, some studies have shown that reactive astrocytes may increase the activities of *γ*-secretase and β-secretase, leading to an increase in Aβ (Ourdev et al., 2015). Thus, there is a vicious cycle between the activation of astrocytes and the pathological effects of Aβ. The aforementioned Aβ-induced damage in the brain is shown in Figure 3.

## 5 Effects of APP and its breakdown product on Aβ production in NMJs

The NMJ acts as a bridge between the muscle and the brain and can transmit the information received by nerve endings to the target muscle to complete the excitation–contraction coupling process (Nishimune and Shigemoto, 2018). The specific excitation transmission process is as follows. The excitation of motor nerve cells opens calcium channels, which enter nerve cells and promote the release of synaptic vesicles, which release acetylcholine into the synaptic cleft. Subsequently, acetylcholine binds to acetylcholine receptors in the motor endplate, allowing cation channels in the postsynaptic membrane to open. This causes changes in the permeability of the cell membrane to potassium and sodium ions. It causes sodium ions to flow in and potassium ions to flow out, resulting in depolarization and the formation of an endplate potential, which spreads to the whole muscle fiber and the interior of the muscle fiber through the transverse tube system and finally causes muscle fiber contraction (Rodríguez Cruz et al., 2020).

APP at the NMJ may be a key factor in the interplay between sarcopenia and AD. Under normal conditions, APP supports synapse formation and cell adhesion, facilitating effective communication between neurons and muscle cells, which is essential for muscle contraction (Zheng and Koo, 2006). Research suggests that APP may regulate neuronal excitability through Kv7 channels, with an abnormal reduction in APP family proteins causing memory deficits and reduced synaptic plasticity at both presynaptic and postsynaptic levels. Selective inhibition of APP expression in excitatory neurons after birth in mice results in impaired memory and synaptic plasticity (Lee et al., 2020). Abnormal deposition of APP at the NMJ can lead to neurodegenerative changes, reducing muscle contraction efficiency. In sarcopenia patients, this may lead to further decreases in muscle mass and strength (Wu et al., 2023). The overexpression of APP in muscle has been linked to mitochondrial abnormalities, resulting in impaired calcium release and muscle contraction force (Boncompagni et al., 2012). These findings suggest that APP abnormalities may damage muscle, increasing the risk of sarcopenia. For example, muscular Swedish mutant APP not only leads to sarcopenia-like deficits but also contributes to AD. In AD patients, APP-associated NMJ damage may exacerbate dual impairments in cognitive and muscle function (Iyer et al., 2021). Furthermore, studies in TgAPP swe HSA mice (a model with muscle-specific expression of APP swe) revealed sarcopenia-like muscle damage, age-related brain pathology, and behavioral abnormalities (Pan et al., 2022). Additionally, research has indicated that APP may interact with myostatin via shared downstream pathways, such as the TGF-β/Smad pathway, influencing both neuronal and skeletal muscle cell functions. Myostatin has been associated with cognitive decline in AD animal models (Lin et al., 2019). Thus, APP dysfunction emerges as a potential common risk factor for both muscle and brain degeneration, although further investigation is needed to elucidate the precise underlying mechanisms involved.

It has been suggested that muscle atrophy or weakness that occurs during aging results from denervation of muscle fibers (Larkin et al., 2011). Recent studies have shown that the cause of sarcopenia may be damage to the NMJ caused by Aβ, leading to a reduction in cholinergic innervation (Torcinaro et al., 2021). Studies have also further supported this point. Yuan et al. (2017) reported increased levels of Aβ in the spinal cord of a TgCRND8 double-mutant AD mouse model, which correlated with motor function deficits. Increased Aβ42 has also been detected in the spinal cord of patients with ALS (Calingasan et al., 2005). Xu et al. (2022) selected young and aged female 3xTgAD mice as subjects. Compared with the control group, aged female 3xTg-AD mice not only presented increased APP, soluble Aβ40 and tau in the brain but also in the spinal cord. In addition, NMJ morphology was altered in aged female 3xTgAD mice compared with age-matched controls, with significant denervation and fragmentation, and the percentage of fragmentation reached 30%, which was significantly greater than that in their age-matched wild-type counterparts. Most importantly, NMJ function was significantly reduced in aged 3xTgAD mice compared with wild-type mice, with a 17% decrease in neural priming (Xu et al., 2022). These findings demonstrate that high levels of Aβ in motor neurons alter the NMJ structure and function, possibly to the extent of denervation, and lead to loss of muscle mass and contractile function. In addition, denervation can also cause mitochondrial damage, leading to muscle degeneration. Muller et al. (2007) severed the sciatic nerve and reported a nearly 30-fold increase in ROS production in muscle mitochondria 7 days after denervation (Muller et al., 2007). Thus, increased ROS production in mitochondria may be a common factor in the mechanism of denervation-induced atrophy. However, the literature shows only the effects of Aβ on the structure and morphology of NMJs and subcellular organelles, and the molecular signaling pathways involved in the regulation of inflammation, oxidative stress, autophagy, etc., have not been explored in depth.

By comparing PI3K pathway activity in 15-day-old flies for each Aβ peptide, the synaptic toxic effects of each Aβ peptide were found to persist in NMJS-overexpressing PI3K. These findings suggest that a direct relationship between Aβ and the PI3K/Akt/GSK3 pathway is central to Aβ-induced synaptic dysfunction and that Aβ40, and possibly Aβ42, does not lead to synapse removal but prevents new synapse formation (López-Arias et al., 2017). In addition, LRP4 (APOE receptor), a MuSK receptor, and APP are essential for the development of special synapses formed between motor neurons and muscles, which are required for the proper formation and function of NMJs (Wang et al., 2005). Excessive APP misdigestion and Aβ production not only disrupt LRP4 binding to agrin (an aggregation protein produced by motor neurons), resulting in failure of Musk activation, thereby inhibiting acetylcholine receptor aggregation in the membrane (acetylcholine receptor aggregation is a key step in NMJ) but also blocking the binding of agrin to APP. This impairs the formation and development of neuromuscular junctions and hinders synaptic differentiation (Choi et al., 2013).

**Figure 4:**
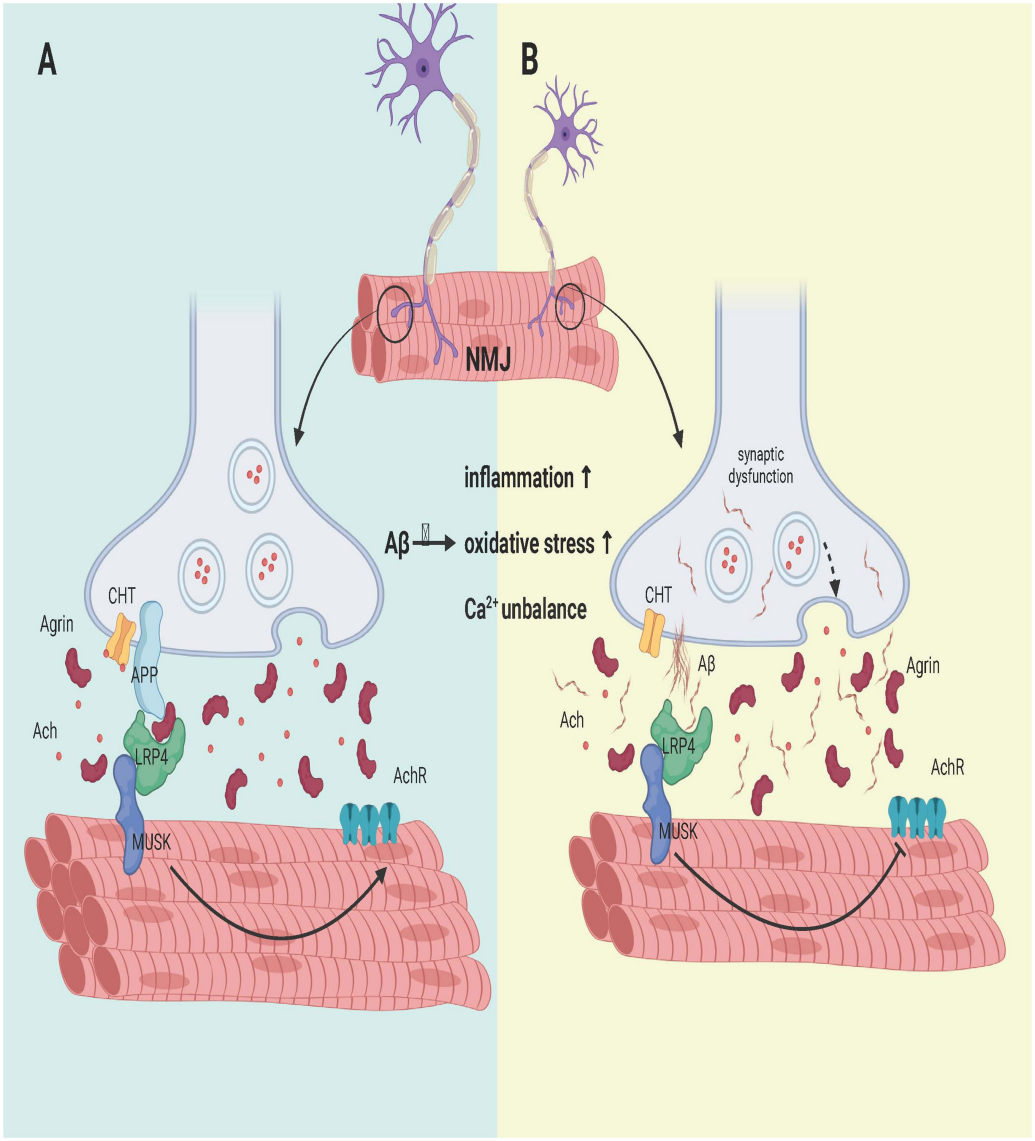
Effect of Aβ on the NMJ formation process. (A) APP interacts with LRP4 and agrin to regulate NMJ formation. APP interacts with LRP4 and activates Musk. The synergistic binding of APP and agrin to LRP4 further strengthens the interaction between LRP4 and APP in muscle, promoting synaptic differentiation and the maintenance of postnatal NMJs. (B) APP hydrolysis leads to the failure of LRP4 binding to APP and the collapse of its domain, which fails to activate Musk and reduces AChR aggregation on the sarcolemma, leading to muscle fiber denervation. Moreover, it affects the development of the NMJ, and the degeneration of the NMJ leads to a reduction in CHT targeting. In addition, the activation of classical inflammation, oxidative stress and calcium ion imbalance by Aβ may also be a potential signaling pathway, but more experimental evidence is needed to confirm these findings. Created with BioRender.com.

In addition, excessive APP decomposition can also cause damage to NMJs. APP deficiency leads to CHT localization defects and decreased presynaptic CHT activity, which ultimately leads to NMJ loss (Wang et al., 2007). APP/APLP2 dko mice exhibit more severe disruption of the NMJ and reduced CHT targeting, which controls cholinergic synaptic transmission rates in the NMJ and CNS, than do APP/APLP2 dko mice (Wu et al., 2023). In addition, studies in which APP is removed from presynaptic motor neurons and postsynaptic muscles have shown that postsynaptic APP depletion also leads to defects in spontaneous vesicle release (Wang et al., 2009) as shown in Figure 4.

## 6 Effects of Aβ in muscle

Aβ can not only affect the brain and NMJ but also exist in muscles and causes damage. For example, Aβ42 deposition in the muscles of autopsied patients was reported by Cox et al. (2011). Increased Aβ levels were also observed in APP/PS1 mice (Schuh et al., 2014). In the following section, we discuss the effects of Aβ on muscle cells and muscle satellite cells.

### 6.1 Effects of Aβ on muscle cells

The accumulation of Aβ can cause an inflammatory reaction in muscles, leading to muscle atrophy and loss of muscle mass. Studies have shown that APP and Aβ aggregates in muscle colocalize with the inflammatory molecules CXCL-9, MHC-I, and IL-1β, suggesting that Aβ must have some connection with inflammation (Greenberg, 2009). Numerous studies have shown that the expression of proinflammatory chemokines and cytokines, such as the interleukin IL-1β, is upregulated in sarcopenia patients, leading to inflammation. In an inflammatory environment, skeletal muscle fibers are directly damaged (Greenberg, 2009). There is a mutual promoting relationship between IL-1β and Aβ (Tu and Li, 2023). In other words, the inflammatory environment can promote the overexpression of Aβ, and high expression of Aβ can also exacerbate the inflammatory response, resulting in muscle fiber damage (Fanlo-Ucar et al., 2024). With increasing APP hydrolysate Aβ42 in muscle, the phosphorylation of tau protein in skeletal muscle not only increases but also accelerates pathology and produces motor defects (Kitazawa et al., 2006). With the increased expression of Aβ in muscle, an inflammatory response is induced, and the expression of TGF-β is upregulated. TGF-β is associated with a number of muscle atrophy pathway-related proteins (Ismaeel et al., 2019). The upregulation of TGF-β leads to the activation and phosphorylation of the transcription factors Smad2 and Smad3 and induces the upregulation of the specific ubiquitin ligase MuRF1, which inhibits skeletal muscle protein folding and ultimately leads to the loss of muscle mass (Sartori et al., 2009). In addition, excessive accumulation of Aβ in muscle can also increase myostatin expression in the inflammatory environment (Starck and Sutherland-Smith, 2010). MSTN is a member of the TGF-β superfamily. Its overexpression not only inhibited the AKT/mTOR axis (Morissette et al., 2009) and the IGF-1-mediated increase in myotube diameter but also activated the activities of the transcription factors Smad2, Smad3 and Foxo (Morissette et al., 2009), leading to myofibrin degradation (Lee H. K. et al., 2012).

The accumulation of Aβ also damages muscle by disrupting Ca^2+^ homeostasis and affecting mitochondrial function. The accumulation of Aβ in cells leads to the dysregulation of calcium ions (Lopez and Shtifman, 2010), and long-term increases in calcium ion levels in cells can impair muscle function (Bartkiewicz et al., 2024). The accumulation of Aβ can reduce the activation threshold of the calcium uniporter on the mitochondrial membrane, which causes more calcium to enter the mitochondria and overload the mitochondrial matrix, leading to mitochondrial matrix swelling, increased mitochondrial permeability, mitochondrial outer membrane rupture and the disappearance of the mitochondrial membrane potential. In addition, myoblasts treated with β-amyloid fibrils exhibit more oxidative damage than myoblasts treated with insulin fibrils do (Jayaraman et al., 2008). With the continuous increase in ROS, the RYR receptor on the sarcoplasmic reticulum calcium channel is fully oxidized, and oxidized RYR1 can increase calcium channel activity, thereby mediating calcium leakage (Shtifman et al., 2010). The continuous outflow of calcium from the sarcoplasmic reticulum leads to the accumulation of calcium in mitochondria, resulting in a vicious cycle. Moreover, the autophagy system that acts on mitochondria starts to eliminate some damaged mitochondria, but the ultrastructure of mitochondria is also destroyed (Xia et al., 2021). The accumulation of Aβ can also lead to a significant decrease in TCA cycle activity and a gradual increase in glucose metabolism from aerobic to anaerobic conditions, resulting in an increase in lactate and a decrease in pH. A more acidic cell-matrix environment leads to a decrease in the affinity of calcium pumps in the sarcoplasmic reticulum for calcium, thereby reducing the rate of calcium uptake by the sarcoplasmic reticulum. Eventually, the excitation–contraction coupling of muscle fibers is destroyed, and symptoms of muscle weakness appear (Lopez and Shtifman, 2010).

Excess Aβ causes proteasomal and lysosomal dysfunction. The main role of the proteasome is to degrade proteins that are not needed or damaged by the cell. The overexpression of Aβ inhibits proteasome activity in muscle fibers, which leads to the accumulation of misfolded potentially cytotoxic proteins that cause muscle damage (Fratta et al., 2005). In addition to its effect on the proteasome, APP can also cause damage to lysosomes. Normally, APP is generated in the endoplasmic reticulum and enters the plasma membrane via secretory vesicles after the maturation stage. APP can enter endosomes through clathrin-dependent endocytosis, followed by transport to lysosomes for degradation or recycling to the cell surface. However, if too much APP is produced, it will burden the lysosomes and cause dysfunction. Blocking autophagy prevents the degradation of APP and other harmful substances, and the accumulation of Aβ may occur, which leads to vacuolization and lipid droplet accumulation in muscle and eventually impairs muscle quality and function (Jiang et al., 2014).

### 6.2 The effect of Aβ on muscle satellite cells

**Figure 5:**
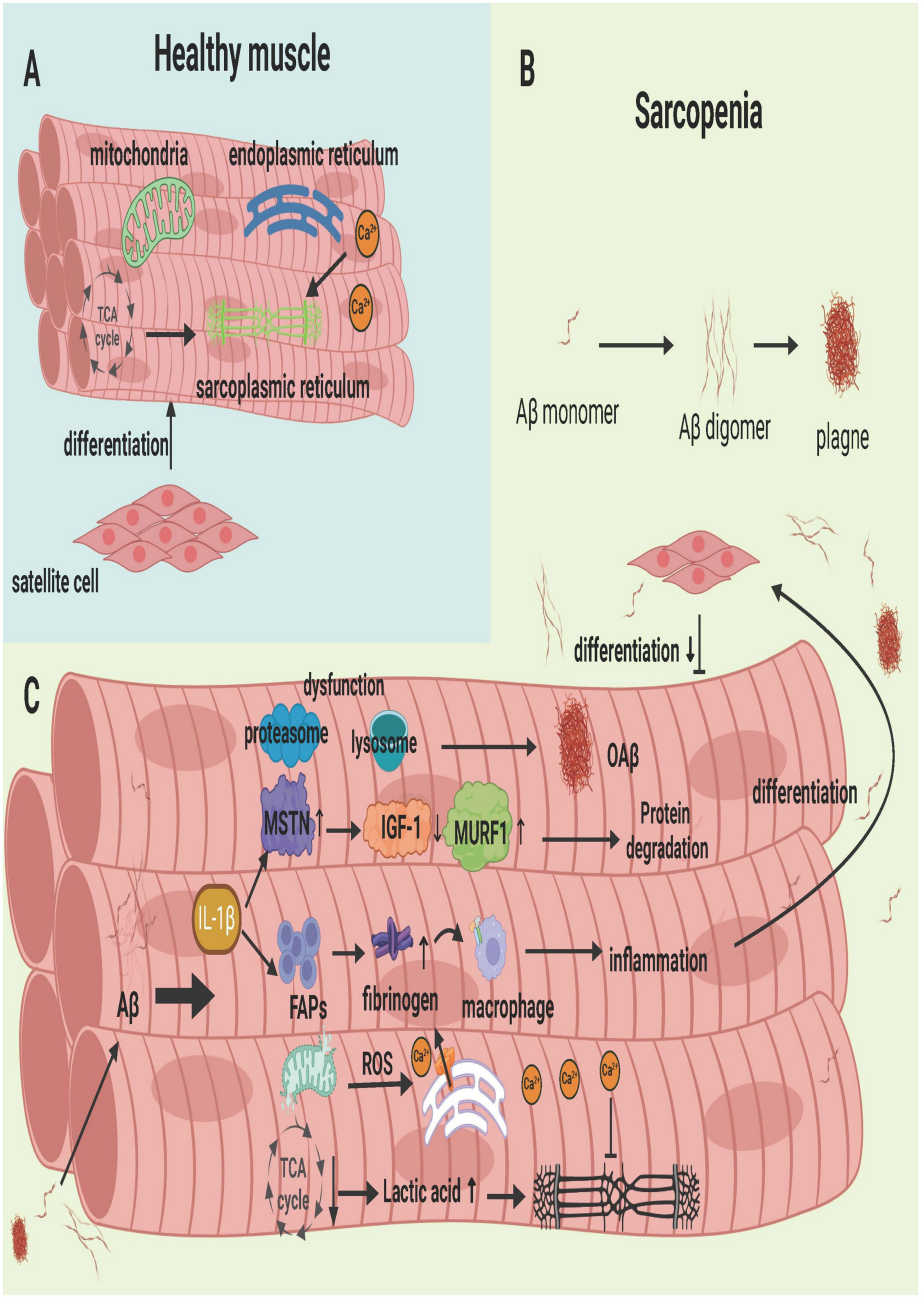
Aβ deposition in Skeletal Muscle in Sarcopenia. (A) Healthy skeletal muscle, where satellite cells retain their differentiation capabilities, and the mitochondria, endoplasmic reticulum, and sarcoplasmic reticulum function normally. (B) Process of single-stranded Aβ aggregating into oAβ. (C) The series of physiological and biochemical reactions resulting from Aβ deposition in muscle. These reactions impair the normal function of cellular organelles, leading to muscle cell damage and the inhibition of satellite cell proliferation and differentiation. Created with BioRender.com.

SCs, also known as myogenic stem cells, are stem cells with the ability to proliferate and differentiate in adult skeletal muscle and are essential for maintaining muscle repair and regeneration (Careccia et al., 2023). When skeletal muscle is injured, SCs proliferate and then differentiate into myoblasts with fusion ability under the action of M1 and M2 macrophages to repair and replace damaged muscle (Dhawan and Rando, 2005). Growth factors can promote the proliferation and differentiation of SCs. Among them, MyoD growth factor regulates the ability of SCs to release the paired box transcription factor Pax7 (Zammit et al., 2006), which is required for muscle satellite cell differentiation, and some of these SCs maintain Pax7 expression and return to the quiescent state to replenish the SC pool. However, excessive accumulation of Aβ leads to an increase in myostatin, which inhibits the synthesis of SCs (Meriggioli and Roubenoff, 2015). The effect of Aβ on SCs is mainly to destroy the cellular environment of Scs, thereby interfering with the repair effect of SCs on muscle. When there is too much Aβ in muscle, skeletal muscle is damaged, leading to inflammation. Proinflammatory cytokines such as TNF-*α*, IL-6 and IL-1β are abundantly expressed. In the early stage of the disease, these proinflammatory cytokines promote myoblast proliferation and Pax7 expression, but when myoblasts infiltrate the inflammatory environment for a long period of time, they inhibit myogenic cell fusion and impair insulin-induced myoblast protein synthesis. Moreover, it promotes SC senescence in the process of repeated degeneration and replication and hinders the myogenic differentiation of SCs (Bigot et al., 2008; Wang et al., 2008). When SCs become dysfunctional, progressive muscle diseases such as sarcopenia and IBM often occur. In addition, the inflammatory environment caused by excessive Aβ accumulation increases the cytokines and cells responsible for the extracellular matrix (FAPs), which leads to abnormal deposition of the extracellular matrix in skeletal muscle and muscle fibrosis (Lemos et al., 2015). Fibrinogen deposits can also bind to Mac-1 receptors on M1 phagocytes to exacerbate the inflammatory response (Vidal et al., 2008), which is not conducive to the timely repair of injured muscles by SCs as shown in Figure 5.

Therefore, the accumulation of Aβ can not only directly impair the function of neurons but also lead to the abnormal activation of microglia and stellate glial cells. This promotes an increase in proinflammatory factors and ROS, causing damage to the brain. Finally, Aβ leads to the occurrence and development of AD. Aβ can also inhibit the connection between neurons and muscles through its effect on the NMJ, leading to muscle atrophy and other lesions. In addition, the accumulation of Aβ can also lead to a series of reactions, such as an imbalance in skeletal muscle calcium homeostasis, differential expression of proteases, dysfunction of lysosomes, and dysfunction of the function of muscle satellite cells, which can damage muscle fibers and eventually lead to sarcopenia. In summary, numerous studies indicate that Aβ, as the cleavage product of APP, plays a pivotal role in the biological mechanism of sarcopenia and AD comorbidity and may be a potential core intervention target for myocerebral comorbidity.

## 7 Therapy

### 7.1 Drug therapy

#### 7.1.1 Drug treatment for AD

Initial AD treatments are broadly categorized by mechanism into two main classes: acetylcholinesterase inhibitors and NMDA receptor antagonists (Passeri et al., 2022). Acetylcholinesterase inhibitors, including donepezil, galantamine, and rivastigmine, enhance cognitive function in mild to moderate AD patients by increasing acetylcholine levels in the brain through cholinesterase inhibition (Sutton et al., 2024). Elevated glutamate levels may induce neurotoxicity, leading to neuronal death. NMDA receptor antagonists, such as memantine, mitigate neuronal damage by modulating glutamate levels and are suitable for moderate to severe AD (Liu et al., 2019). Despite their widespread use, these drugs offer limited efficacy and do not address the underlying neurodegenerative changes. Consequently, research is increasingly focused on earlier or more “upstream” AD pathology targets. Current disease-modifying therapies (DMTs) aim to alter AD progression by preventing or delaying disease development, reversing or repairing pathological changes, and include strategies that target Aβ deposition, tau hyperphosphorylation, neuroinflammation, neuronal survival, and synaptic function (Wang et al., 2024). For example, antiamyloid drugs (e.g., aducanumab and lecanemab) clear amyloid plaques (Perneczky et al., 2024), whereas anti-tau treatments are in clinical trials to inhibit neurofibrillary tangle formation (Ye et al., 2024). Neuroprotectants, such as huperzine A and ginkgo biloba extracts, may also support cognitive improvement (Patel et al., 2024).

Exploring AD immunotherapy from the perspective of amyloid-β (Aβ) is a prominent research focus. Clinical research faces the dual challenges of inhibiting Aβ formation and aggregation while curbing related inflammatory responses and oxidative stress cascades. These objectives could mitigate the onset and progression of AD, positioning this approach as both a critical and complex area of investigation.

One therapeutic approach to prevent the excessive production of Aβ is through inhibiting the cleavage of APP. This category includes drugs that target *γ*-secretase (Egan et al., 2019) and BACE1 inhibitors (Ghosh, 2024). γ-Secretase plays a critical role in cleaving APP into Aβ, and by inhibiting its activity, Aβ production can be reduced. However, clinical trials have shown that γ-secretase inhibitors, such as semagacestat (Eli Lilly) and avagacestat (Bristol-Myers Squibb) (Tagami et al., 2017; Simutis et al., 2018), not only lack efficacy but also induce severe side effects, ultimately leading to the termination of these trials. A possible explanation for this outcome is that γ-secretase acts on other physiological substrates, such as Notch, impacting essential biological functions (Tolia and De Strooper, 2009).

Another target in drug development is BACE1 inhibitors. BACE1 is a transmembrane aspartyl protease responsible for cleaving APP at the β-site; BACE1 and γ-secretase sequentially process APP, thereby increasing Aβ production and release in the brain. Preclinical studies have suggested that BACE1 inhibitors are promising for reducing brain Aβ levels and even delaying AD progression. For example, verubecestat and atabecestat (Egan et al., 2018; Koriyama et al., 2021) demonstrated such potential. However, clinical trials have revealed that these drugs do not effectively slow cognitive decline. One potential reason is that, while BACE1 inhibitors reduce Aβ production, they fail to inhibit the compensatory rise in other secretases, leading to an increase in APP metabolites. Furthermore, they do not act directly on preexisting Aβ deposits (Chagas Monteiro et al., 2023). Studies also indicate that BACE1 knockout mice exhibit seizure symptoms, highlighting alterations in neuronal activity and neurodegeneration and emphasizing the essential role of BACE1 in maintaining fundamental brain functions (Hu et al., 2010).

Additionally, aggregation of the APOE protein may be a significant factor contributing to Aβ accumulation and pathogenesis. Researchers at the Technical University of Munich have reported that APOE protein aggregation could be a critical process in Aβ plaque formation, particularly in carriers of the APOE4 allele. APOE aggregates can induce Aβ fibrillization and accelerate plaque deposition, suggesting a novel perspective for AD treatment (Kaji et al., 2024).

Among the most prominent therapeutic approaches are Aβ-targeted immunotherapies. Currently, three anti-Aβ monoclonal antibodies have been approved: aducanumab, lecanemab, and donanemab. Aducanumab is a selective, human-derived monoclonal antibody that targets aggregated Aβ. It selectively binds to amyloid Aβ in the brains of AD patients and activates the immune system to clear Aβ deposits from the brain (Sevigny et al., 2016). In the EMERGE clinical trial, a high dose of 10 mg/kg aducanumab was shown to improve cognitive function, with PET scans indicating significant reductions in brain amyloid levels among patients receiving the high dose (Budd Haeberlein et al., 2022). Consequently, on June 7, 2021, the FDA approved this antibody for the clinical treatment of AD. However, during treatment, aducanumab was associated with amyloid-related imaging abnormalities, including edema (ARIA-E) and/or microhemorrhage (ARIA-H), as detected on MRI scans (Budd Haeberlein et al., 2022). Lecanemab, a humanized mouse monoclonal antibody, has high affinity for soluble Aβ protofibrils (van Dyck et al., 2023). In June 2023, the FDA approved lecanemab for treating mild cognitive impairment and mild dementia due to AD. Compared with placebo, lecanemab reduced clinical deterioration by 27% over 18 months, with improvements in all secondary outcomes, including cognition, activities of daily living, quality of life, and caregiver burden. Aβ-PET imaging confirmed a marked reduction in Aβ, supporting its clinical benefits (Swanson et al., 2021). However, other studies indicate that lecanemab treatment may cause cerebral hemorrhage in patients receiving anticoagulants, underscoring the need for further safety data to validate its efficacy and safety (Pfeifer et al., 2002). Donanemab is a monoclonal antibody that binds to Aβ modified at the N-terminal position by pyroglutamate, promoting rapid clearance of amyloid deposits. On July 2, 2024, the FDA approved donanemab for treating early symptomatic AD. Studies evaluating donanemab’s efficacy and safety revealed a 35% reduction in cognitive decline and a 36% reduction in clinical progression over 18 months. Additionally, it reduced the decline in daily living abilities by 40% and lowered the risk of disease progression by 39%. Aβ plaques were reduced by 84% in the donanemab group (Mintun et al., 2021; Sims et al., 2023).

Research into AD treatments continues, with an emerging focus on enhancing drug efficacy through nanoparticle-assisted delivery. This approach aims to improve targeting precision and bioavailability. These drugs are engineered with specific ligands that bind to receptors on brain cells, ensuring accurate delivery to target areas, minimizing systemic side effects, and enabling controlled release of therapeutic doses. This method also holds promise in addressing the challenge of drug passage across the blood–brain barrier (Hu et al., 2023). Such interdisciplinary therapeutic strategies are likely to become a significant trend in future AD treatment development.

#### 7.1.2 Drug treatment for sarcopenia

Medical treatments for sarcopenia include growth hormones, anabolic or androgen steroids, selective androgen receptor modulators, protein anabolic agents, appetite stimulants, myostatin inhibitors, agonist type II drugs, β-blockers, angiotensin-converting enzyme inhibitors, and troponin activators, but their efficacy is not ideal. For example, growth hormone increases muscle protein synthesis and increases muscle mass, but it does not improve muscle strength or function (Sakuma and Yamaguchi, 2012). The effect of anabolic steroid supplementation differs between the sexes. In contrast to the increase in body weight and lean mass in men, the increase in body weight in women is due mainly to an increase in fat mass (Meriggioli and Roubenoff, 2015). Drugs that block Aβ formation in skeletal muscle, which were not identified in the current review, may have similar effects on AD treatment as described above. This may also be because research on the inhibition of Aβ formation in skeletal muscle is in its infancy, which is a topic of increased interest and is a future research direction.

In the present review, no drugs that block Aβ formation in skeletal muscle were identified. This may be due to the similar reasons mentioned above in the treatment of AD or because the inhibition of Aβ formation in skeletal muscle is still being studied, which is a topic of interest for future research.

### 7.2 Sports therapy

In view of the current situation and problems such as the poor efficacy of drug intervention for Aβ, we focused on exercise intervention, which is an old but new method. Aerobic exercise can improve the body’s ability to use oxygen and enhance heart and lung function. It also boosts immunity, reducing the risk of infections and inflammatory diseases. At the same time, it can promote blood circulation to the brain to improve brain function and improve cognitive function and learning ability (Fleg, 2012). Resistance exercise can not only improve muscle strength but also improve brain function and stimulate the continuous growth of brain cells (Kirkman et al., 2022). It can also reduce anxiety and depression. Therefore, both aerobic and resistance exercise can have beneficial effects on muscles and the brain. In this review, we discuss the effects of different exercise modes on Aβ in the muscle and brain.

#### 7.2.1 The effects of exercise on Aβ in the brain

Exercise is associated with a reduced risk of AD (Buchman et al., 2012). In animal models of AD, commonly used interventions fall into two categories: voluntary exercise and forced exercise, both of which have inhibitory effects on Aβ formation (Andrade-Guerrero et al., 2023b). For example, in a study in which voluntary exercise was used as an intervention, Andrade-Guerrero et al. (2023a) conducted voluntary wheel running for 2 h a day and 5 days a week for 3 months in 40 10-month-old 3XTgAD female mice. The results revealed that the content of Aβ in the brains of exercised mice was reduced and that cognitive function and NVU function were improved. Previous studies in which 5xFAD Tg male mice of different ages (6, 4, and 8 months) were subjected to voluntary wheel running for 6 weeks in an enriched environment revealed that it not only restored neurogenesis and reversed memory deficits but also reduced Aβ deposition and the generation of apoptotic cells and promoted the generation of phagocytic microglia (Ziegler-Waldkirch et al., 2018). Kim et al. (2019) selected 10 3-month-old 3xTgAD male mice to exercise on a treadmill 5 days per week for 12 weeks and reported that the exercise group presented reduced levels of Aβ plaques and neuroinflammation and improved mitochondrial function, neurogenesis, and spatial memory compared with the control group. Stair climbing exercise 3 days a week for 12 weeks in 30 9-month-old 3xTg mice improved cognitive performance, reduced amyloid plaques and tau phosphorylation, and inhibited the activation of microglia and astrocytes (Liu et al., 2020). In addition, irisin is known to protect the hippocampus of mice that are more susceptible to AD by inhibiting Aβ accumulation (Jin et al., 2018). It has also been shown that 5 weeks of swimming can improve the normalization of hippocampal irisin expression in AD patients, accompanied by a reduction in Aβ protein levels and the inhibition of tau protein phosphorylation (Hegazy et al., 2022). Indeed, low PGC-1*α* expression is known to cause Aβ accumulation in the brains of AD patients (Jin et al., 2018). Owing to the activation of PGC-1α (an upstream activator of the irisin precursor FNDC5) after exercise, not only APP cleavage but also Aβ formation may decrease.

#### 7.2.2 The effect of exercise on Aβ in muscle

Exercise not only reduces Aβ deposition in the brain but also decreases the Aβ content in skeletal muscle. In one study, 20 13-month-old NSE/ps2m Tg AD mice were divided into a control Tg group, an exercise Tg group, and a control non-Tg group. The exercise group performed nontilt treadmill exercise for 60 min per day, 5 days per week, for 12 weeks. The running time (from 30 to 60 min) and treadmill speed (from 5 to 12 m/min) increased progressively within 12 weeks. Western blot analysis of the plantaris muscle of the mice revealed that the expression of Aβ42 in the skeletal muscle of the Tg mice subjected to treadmill exercise was significantly lower than that in the skeletal muscle of the Tg mice in the control group. Moreover, tissue staining with Congo red revealed diffuse amyloid deposition in Tg mice, but its distribution was significantly reduced in Tg mice subjected to 12 weeks of treadmill exercise training (Yook and Cho, 2017). In addition, 12-month-old NSE/PS2m Tg mice were designed to exercise on a treadmill. Western blot analysis revealed that the concentration of Aβ in the skeletal muscle of the exercise group was lower than that in the nonexercise group, and the immunoreactive particulate deposits in the thigh muscle cells of the exercise group were lower than those in the control group (Cho et al., 2003). These findings suggest that treadmill exercise reduces Aβ protein levels in skeletal muscle. In addition, resistance exercise can improve mitochondrial function by reducing the accumulation of Aβ in muscle to alleviate muscle damage. Some studies have shown that weight-bearing ladder climbing for 9 weeks improved mitochondrial quality control and oxidative capacity and reduced muscle damage in mice. Resistance exercise was shown to reduce Aβ accumulation and increase mitochondrial oxidative capacity by activating sirtuin 3 signaling to upregulate superoxide dismutase-2, catalase and citrate synthase (Koo et al., 2019).

**Figure 6:**
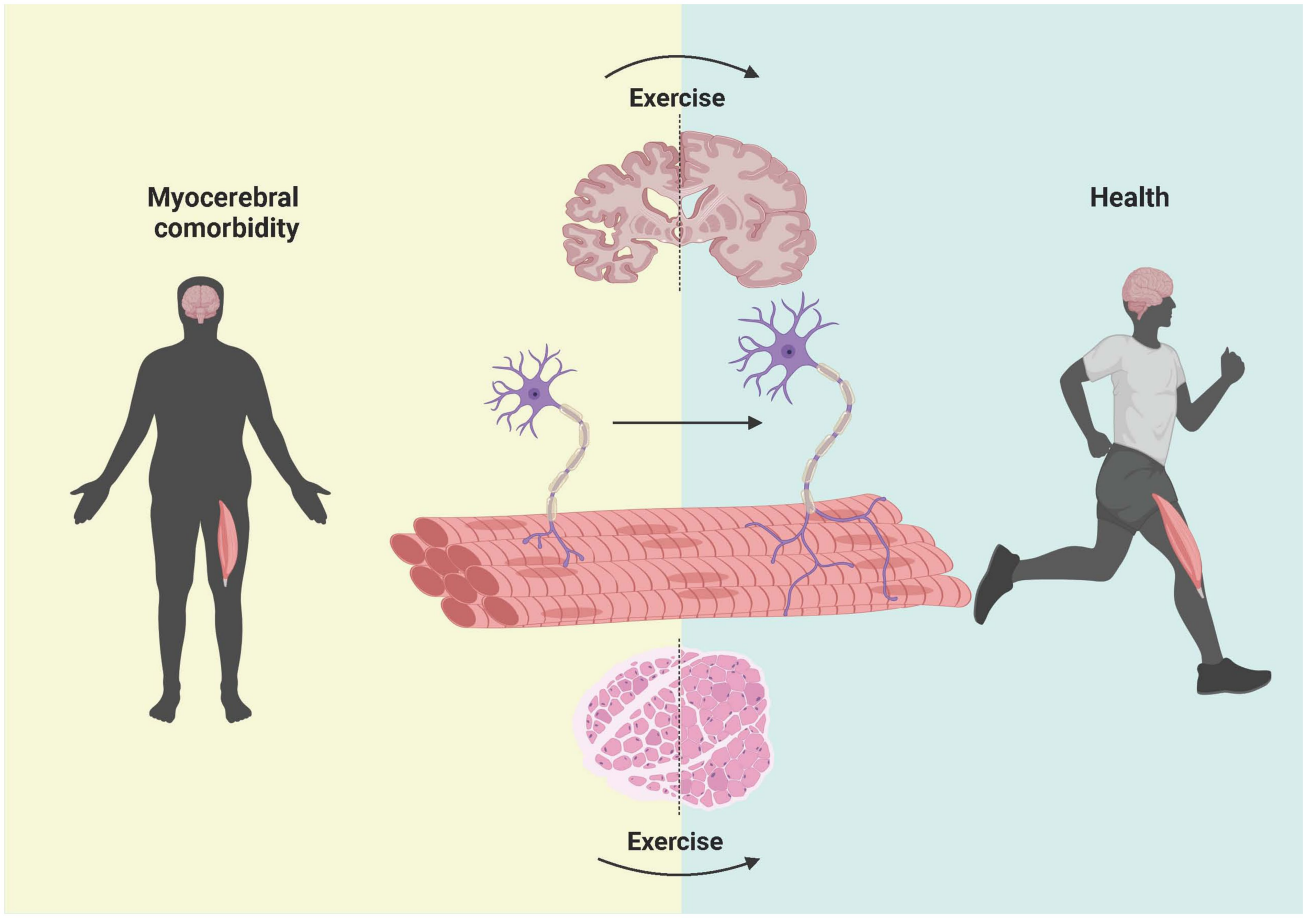
Effect of exercise intervention on Aβ in the brain, NMJ, and muscle in patients with myocerebral comorbidities. The left side shows the brain, NMJ, and muscle of patients with myocerebral comorbidities. After the deposition of Aβ was reduced through exercise, the shape and size of the brain and muscle of the patients changed significantly and gradually changed in the direction of the healthy people on the right side. Previous studies have shown that exercise can improve the integrity of the NMJ in patients. However, whether reducing Aβ through exercise can lead to improvements in NMJ integrity and the underlying mechanisms need to be further studied. Created with BioRender.com.

In conclusion, the protein levels of Aβ in both the brain and skeletal muscle can be decreased by exercise, and exercise has good efficacy and few side effects compared with drug treatment. The choice of exercise method, whether it is treadmill aerobic exercise or weight-bearing ladder climbing resistance exercise, plays a certain role. We are optimistic that, according to the results of animal experiments, aerobic or resistance exercise, such as jogging, variable-speed running, and stair climbing, can be emulated in humans to reduce the adverse effects of Aβ on muscle–brain comorbidity, but many experiments are still needed to confirm these findings as shown in Figure 6.

## 8 Summary and prospects

In this review, we focused on Aβ, the cleavage product of APP, and comprehensively described the common pathological mechanism and targeted therapy of AD and sarcopenia. AD and sarcopenia share similar pathological processes, such as oxidative stress, inflammation, aging, and mitochondrial dysfunction, in NMJs, neurons, and skeletal muscle cells. As a protein that is present in both muscle and neurons, Aβ plays an important role in the occurrence and development of muscle–brain comorbidities. However, the molecular mechanisms of Aβ in muscle and brain pathology and functional impairment are still poorly understood, especially in muscles and NMJs. In existing studies, the source and destination of Aβ are generally well studied in the brain, but few studies have focused on the muscle. Whether there are differences needs further study. Considering the evidence collected in this review, we are confident that one of the common mechanisms of muscle–brain comorbidity is the accumulation of Aβ in the brain and muscle, which disrupts the homeostasis of the body and leads to sarcopenia and AD. In the future, we will focus on the spatiotemporal expression characteristics of Aβ in skeletal muscle and NMJ and the important biological cascades induced by Aβ. In addition, there is a lack of targeted drug therapies for AD and sarcopenia based on Aβ. We believe that effective exercise interventions may play an important role in reducing the accumulation of Aβ, but the intrinsic mechanism and the reasonable choice of exercise paradigm need to be further explored. In addition, most current drug therapies and exercise therapies are aimed at inhibiting the formation of Aβ or promoting its deaggregation and decomposition. However, Aβ can cause various cascade reactions and damage to the brain, skeletal muscle and NMJs, such as oxidative stress, chronic inflammation, and autophagy, which are common causes of the occurrence and development of muscle–brain comorbidities. The key molecular targets in these complex signal transduction pathways are likely to be the targets and hotspots of intervention research, including exercise, which is the direction we are actively exploring now and in the future.

## Glossary

Abbreviation: Complete lexical patterns
ACD: Acidic domain
AD: Alzheimer’s disease
ADL: Activities of daily living
AICD: APP intracellular domain
ALS: amyotrophic lateral sclerosis
AMPAR: α-amino-3-hydroxy-5-methyl-4-isoxazole-propionic acid receptor
APOE4: Apolipoprotein E4
APP: Amyloid precursor protein
AQP4: Aquaporin protein-4
α7-nAchR: α7 nicotinic acetylcholine receptor
ABAD: Amyloid β binding alcohol dehydrogenase
BACE1: β-secretase 1
BBB: Blood–brain barrier
BEC: Brain endothelial cells
BIA: Bioelectrical impedance analysis
CAPPD: Central APP domain
CaSR: Calcium-sensing receptor
CCL12: C-C motif chemokine 12
CD36: Cluster of differentiation 36
CD47: Cluster of differentiation 47
CDK5: Cyclin-dependent kinase 5
CHT: Choline transporter
CNS: Central nervous system
COX-2: cyclooxygenase-2
CSF: Cerebrospinal fluid
CT: Computed tomography
CTF: C-terminal fragment
CUBD: Copper-binding domain
CXCL10: C-X-C motif chemokine ligand 10
CXCL-9: C-X-C motif chemokine ligand 9
DAP12: DNAX activation protein 12
DLB: Dementia with Lewy bodies
Drp1: Dynamin-related protein-1
DXA: Dual-energy X-ray Absorptiometry
EPHB2: Erythropoietin-producing hepatoma cell line B2
EPHA4: Ephrin type a receptor4
ER: Endoplasmic reticulum
EWGSOP: European working group on sarcopenia in the older people
FAPS: Fibroblast adipose progenitor cells
FDA: Food and Drug Administration
FNDC5: Fibronectin type III domain-containing protein 5
FOXO: Forkhead box O
Fyn: Family tyrosine kinase
GFLD: Growth factor-like domain
GLT-1: Glutamate transporter 1
Glu: Glutamate
GLIS: Global leadership initiative on sarcopenia
GRP78: 78-kDa glucose-regulated protein
GSDMD: Gasdermin D
GSK-3β: Glycogen synthase kinase 3β
HSA: Human skeletal actin
HPSGs: HS proteoglycans
HSF-1: Heat shock factor-1
IBM: Inclusion body myositis
IDE: Insulin Degrading Enzyme
IGF-1: Insulin-like growth factor-1
IL-1: Interleukin 1
IL-12: Interleukin 12
IL-17: Interleukin 17
IL-18: Interleukin 18
IL-1β: Interleukin 1β
IL-23: Interleukin 23
IL-6: Interleukin 6
iNOS: Inducible nitric oxide synthase
ISF: Interstitial fluid
KPI: Kunitz protease inhibitor
LDLR: Low density lipoprotein receptor
LiLRB2: Leukocyte immunoglobulin like receptor B2
LRP1: Low density lipoprotein receptor related protein 1
LTD: Long-term depression
LTP: Long-term potentiation
MAFbx: Muscle atrophy F-box protein
MERCS: mitochondria-ER contact sites
MURF-1: Muscle ring finger 1 protein
MAPK: Mitogen-activated protein kinase
mGluRs: Group I metabotropic glutamate receptors
MHC-I: Mouse major histocompatibility complex I
MRI: Magnetic resonance imaging
MSTN: Myostatin
MuSK: Muscle-specific tyrosine kinase
MyoD: Myogenic differentiation
NEP: Neprilysin
NFAT: Nuclear factor of activated t cells
NF-κB: Nuclear factor kappa-B
NLRP3: NOD-like receptor thermal protein domain associated protein 3
NMDAR: N-methyl-D-aspartate receptor
NMJ: Neuro-muscular junction
NVU: Neurovascular unit
oAβ: Oligomeric Aβ
PDK-1: Phosphatidylinositol-dependent kinase-1
Pax7: Paired box 7
PDI: Protein disulfide isomerase
PET: Positron emission tomography
PGC-1α: Peroxisome proliferator-activated receptor gamma coactivator-1alpha
PH: Potential of hydrogen
PrPc: Prion protein cellular
PS1: Progerin 1
PS2: Progerin 2
RAGE: Receptor for advanced glycation end products
RNS: Reactive nitrogen substances
ROS: Reactive oxygen species
RYR: Ryanodine receptor
RYR1: Ryanodine receptor 1
SCs: satellite cells
SMAD2: Recombinant mothers against decapentaplegic homolog 2
SMAD3: Recombinant mothers against decapentaplegic homolog 3
STEP: Striatum-enriched tyrosine phosphatase
SYK: Spleen tyrosine kinase
TCA: Tricarboxylic acid
Tg: Transgenic
TGF-β: Transforming growth factor-β
TLR4: Toll-like receptor 4
TNF: Tumor necrosis factor
TNF-α: Tumor Necrosis Factor-alpha
TREM2: Triggering receptor expressed on myeloid cells 2
VDCA1: Voltage dependent anion channel protein 1
VGLUT1: Vesicular glutamate transporter 1
Vti1a: Vesicle transport through interaction with t-SNAREs homolog 1A
XBP-1: X box-binding protein-1
